# Towards the replacement of wheat ‘Green Revolution’ genes

**DOI:** 10.1093/jxb/eraa494

**Published:** 2021-02-02

**Authors:** Stephen Pearce

**Affiliations:** Department of Soil and Crop Sciences, Colorado State University, 307 University Ave, Fort Collins, USA

**Keywords:** Dwarfing, gibberellin, Green Revolution, Rht, wheat

## Abstract

This article comments on:

**Tang T, Botwright Acuña T, Spielmeyer W and Richards RA**. 2021. Effect of gibberellin-sensitive Rht18 and gibberellin-insensitive Rht-D1b dwarfing genes on vegetative and reproductive growth in bread wheat. Journal of Experimental Botany **72,**445–458.

**First introduced into wheat breeding programs in the 1930s, the *Rht-B1b* and *Rht-D1b* ‘Green Revolution’ alleles remain the most widely used dwarfing genes in today’s wheat varieties. Wheat plants with shorter stems channel a greater proportion of their resources into developing grains, although these alleles also reduce coleoptile length, limiting their utility in dry environments where seeds must be sown deeply. In this issue,** Tang *et al.* (2021) **show in a series of comprehensive field trials that an alternative dwarfing allele, *Rht18*, confers a reduction in height nearly identical to *Rht-D1b*, but has no impact on coleoptile length. These findings highlight the promise of replacing dwarfing alleles as a strategy to optimize wheat height according to the target environment.**

## The value of being short

In agriculture, semi-dwarf plants have several important advantages over their taller, ancestral forms. Most notably, shorter stems pose less competition for limited resources, meaning that a greater proportion of the plant’s photosynthate is partitioned into spike and grain development, improving harvest index and yield in most environments (Youssefian *et al.*, 1992). Furthermore, genetic variants that restrict the rate of cell elongation in the stem, resulting in a shorter plant, mean that growers can fully exploit the benefits of fertilization and irrigation without risking their crop lodging due to excessive height.

Although many genes contribute to plant height, most semi-dwarf crop varieties grown today carry one of a small number of variants that modify the levels or activity of the plant hormone gibberellin (GA). Actively growing tissues contain high levels of bioactive GA, which, among other actions, promotes cell expansion. Many semi-dwarf rice varieties carry a recessive loss-of-function mutation in *GA20ox2* that encodes a critical GA biosynthesis enzyme (Spielmeyer *et al.*, 2002). Because this allele is predominantly expressed in the developing stem, the reduction in bioactive GA levels, and thus its impacts on cell elongation, are most evident in these tissues.

In contrast, the most common dwarfing alleles in wheat are *Rht-B1b* and *Rht-D1b* that encode N-terminally truncated DELLA proteins that impede GA signaling (Peng *et al.*, 1999). These variants originated in the Japanese variety ‘Norin 10’, released in 1935. They were first introduced into US wheat germplasm in the 1950s and subsequently transferred to Norman Borlaug at CIMMYT, where they were integrated into ‘Green Revolution’ varieties that were distributed worldwide (Hedden, 2003). It is remarkable, and a testament to their value for wheat growers, that >80 years after their first use in formal breeding programs these alleles remain such an integral component of wheat variety development. Approximately 63% of cultivars in a worldwide winter wheat panel that were released in the 21st century carry one of these ‘Green Revolution’ alleles (Würschum *et al.*, 2017), while among varieties bred for the Eastern and Central USA, the proportion is >90% (Guedira *et al.*, 2010).

Despite their widespread deployment, wheat varieties carrying these alleles perform less well in warm, dry environments such as in parts of Australia and Mediterranean climates, where limited soil moisture requires that seeds be sown deeply. Because *Rht-B1b* and *Rht-D1b* are expressed in the coleoptile, this tissue is shorter in plants carrying these alleles, reducing the rate of seedling emergence and negatively impacting crop establishment in these environments (Rebetzke *et al.*, 2014). These drawbacks have incentivized the search for alternative dwarfing alleles that might combine an optimal reduction in final plant height without restricting coleoptile and seedling growth. To this end, researchers have identified a number of such alleles and have recently made progress in characterizing their mode of action (Gasperini *et al.*, 2012; Ford *et al.*, 2018; Buss *et al.*, 2020). To help evaluate the potential of these alleles for wheat breeding, carefully designed field experiments will be required. In this issue, Tang *et al.* describe a series of field trials that highlight the promise of *Rht18* as a replacement for the ‘Green Revolution’ alleles.

## Characterizing alternative dwarfing alleles

The *Rht18* semi-dwarf allele has a different mode of action from *Rht-D1b*, and is associated with increased expression of *GA2ox-A9*, which encodes a GA-inactivating enzyme that reduces bioactive GA levels (Ford *et al.*, 2018). Through a comprehensive analysis of the growth dynamics from seedling emergence to maturity, Tang *et al*. show that *Rht18* and *Rht-D1b* confer a near-identical reduction in height in both dryland and irrigated field trials. The authors also found no differential effects on flowering time, spike length, stem density, tiller number, and a host of other traits that are core targets for selection in any breeding program. Crucially, while the *Rht-D1b* allele reduced coleoptile length, there was no shortening of the coleoptile in plants carrying the *Rht18* allele. These findings are highly encouraging, and suggest that replacing ‘Green Revolution’ semi-dwarfing alleles with *Rht18* could be a promising strategy to improve wheat productivity, especially in dry environments where seedling emergence and crop establishment are key considerations for growers. The frequency of *Rht18* among global wheat varieties is increasing, suggesting that this allele is subject to active selection in breeding programs (Würschum *et al.*, 2017).

To gain a better sense of this potential, it will be important to test the impacts of *Rht18* in large, replicated yield trials using independent genetic backgrounds and a more diverse set of environments, especially those with limited moisture. These trials might include an analysis of below-ground traits by leveraging high-throughput root imaging or remote sensing technologies (Atkinson *et al.*, 2019; Yang *et al.*, 2020). Since GA also promotes root cell elongation (Bai *et al.*, 2013), it will be interesting to compare the impact of different dwarfing alleles on root length and architecture, particularly as the frequency and severity of drought events are likely to increase in the coming decades.

## Customizing wheat height for diverse environments

Perhaps most importantly, this study provides further support for the hypothesis that there is nothing intrinsically unique about the ‘Green Revolution’ semi-dwarfing alleles for increasing wheat yields. It appears that any reduction in height, regardless of mechanism, will increase photosynthate partitioning to the grain. This suggests that there may be other excellent candidate alleles among those already described that might similarly be subjected to expanded field testing to evaluate their value to breeding programs and wheat producers (Box 1). These include independent GA-sensitive alleles such as *Rht12* (Sun *et al.*, 2019; Buss *et al.*, 2020) and *Rht25* (Mo *et al.*, 2018), as well as *TEOSINTE BRANCHED 1*, which was recently shown to regulate height and architecture (Dixon *et al.*, 2020).

Box 1. **Mechanisms to reduce wheat height**

At least 21 dwarfing alleles have been described in wheat. Cloning and functional characterization of these genes will improve our understanding of the pathways contributing to height, facilitate searches for additional genetic diversity, and allow breeders to make more informed decisions when combining alleles. The dwarfing genes that have been characterized to date play important roles in hormone biosynthesis and signaling pathways, and confer reduced height through different mechanisms.

**Reduced GA signaling.** The *Rht-B1b* and *Rht-D1b* ‘Green Revolution’ alleles encode truncated DELLA proteins that are insensitive to GA, resulting in constitutive growth repression in the stem. Other natural alleles at these loci confer different degrees of height reduction, including the severe dwarfing allele *Rht-B1c*.

**Removal of GA precursors.** Although the causative variants remain to be isolated, *Rht18*, *Rht14*, and *Rht24* are probably allelic, and all three are associated with increased expression of *GA2ox-A9*. This enzyme removes GA precursors, reducing bioactive GA levels, and thus cell elongation, in the stem. An independent locus, *Rht12*, appears to act in a similar manner, and is associated with increased expression of *GA2ox-A13*.

**Reduced GA biosynthesis.** In rice, recessive, loss-of-function alleles of *GA20ox2*, which encodes a GA biosynthesis enzyme, reduce bioactive GA levels, and thus cell elongation, in the stem. In polyploid wheat, these alleles are unlikely to be selected due to functional redundancy, but could be induced with reverse genetics tools such as TILLING or CRISPR/Cas9.

**Reduced BR sensitivity.** The *Rht8* allele is a widely deployed alternative dwarfing allele common in warm and dry environments. *Rht8* has not yet been cloned, but plants carrying this allele exhibit reduced sensitivity to brassinosteroids.

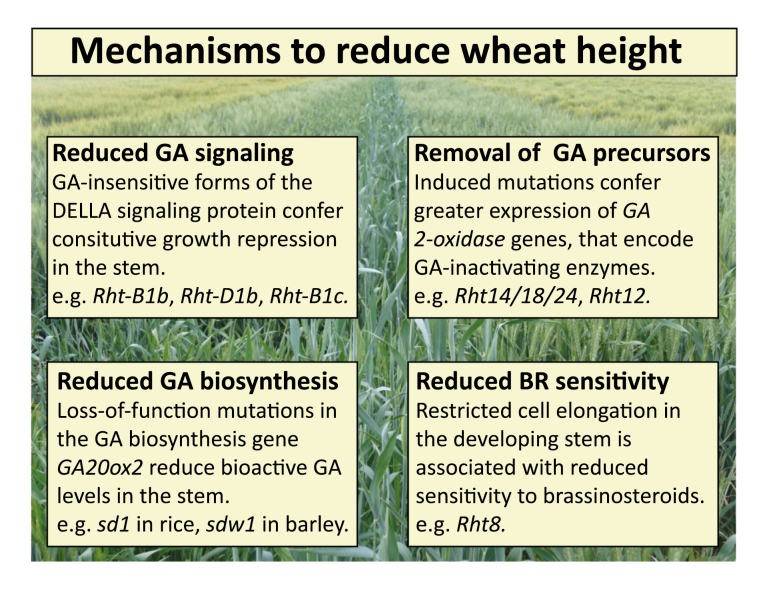

In addition to characterizing known dwarfing loci, wheat researchers now have access to tools such as CRISPR/Cas9 [clustered regularly interspaced palindromic repeats (CRISPR)/CRISPR-associated protein 9] that can enhance allelic diversity by directly engineering genetic variation in specific target genes (Zhang *et al.*, 2020). As well as *Rht18*, at least one other dwarfing locus (*Rht12*) is associated with increased expression of a *GA 2-oxidase* gene (Ford *et al.*, 2018; Sun *et al.*, 2019; Buss *et al.*, 2020). Therefore, it may be possible to develop a range of valuable dwarfing alleles by inducing variation in the *cis*-regulatory regions of members of this multigene family. As we build a mechanistic understanding of the genetic pathways regulating wheat height, reverse genetics tools also provide the opportunity to uncover ‘hidden variation’ in the polyploid wheat genome, by combining loss-of-function alleles in all three homoeologous copies of a target gene (Borrill *et al.*, 2015). One application of this strategy would be to engineer a *GA20ox2* knockout in wheat, replicating the recessive semi-dwarfing alleles in rice that, because of functional redundancy, are unlikely otherwise to be selected.

To help address our urgent need to increase food production, it is imperative that breeders can access broad genetic diversity to fine-tune critical traits (Eshed and Lippman, 2019). By subjecting an expanded set of alternative dwarfing alleles to comprehensive field phenotyping following the example of Tang *et al.*, breeders will have more options to develop optimal allelic combinations for their target environments. Although applicable to most traits, this approach is likely to be particularly valuable for plant height considering its historically outsized impact on wheat yields. Through a combination of genomics analyses and traditional field-based phenotyping and selection, we may soon see the emergence of more semi-dwarf wheat varieties carrying a greater diversity of reduced height genes, either in combination with—or as replacements for—the ‘Green Revolution’ alleles.
